# Supplementary material for: Feeding state functionally reconfigures a sensory circuit to drive thermosensory behavioral plasticity
Source: eLife. 2020 Oct 19;9:e61167. doi: 10.7554/eLife.61167 (PMC7644224; doi:10.7554/eLife.61167)
Supplement: Supplementary file 1. [file elife-61167-supp1.docx]

**Supplementary Table 1.** Strains used in this work.

| **Strain** | **Genotype** | **Source/parent strains** | **Relevant Figures** |
| --- | --- | --- | --- |
| WT | N2 (Bristol) | CGC | 1B, 1C, 1D, 2A, 2B, 3F, 3G, 3H, 3I, 4A, 4D, 4E, S1-1A, S1-1B, S1-1C,  S2B, S2C,  S3E, S3F,  S4-2A,  S4-2C, S4-2D |
| DCR3055 | *wyIs629*[*gcy-8*p::*GCaMP6s* + *gcy-8*p::*mCherry* + *unc-122*p::*gfp*] | (Hawk et al., 2018) | 1E |
| DCR3056 | *olaIs17*[*mod-1*p::*GCaMP6s + ttx-3*p::*mCherry* + *unc-122*p::*dsRed*] | (Hawk et al., 2018) | 1F, 1G, S1-2A, S1-2B |
| PY12205 | *oyEx657*[*odr-1*p::*HisCl1*::*SL2*::*mCherry* + *unc-122*p::*gfp*] | This paper | 2A, S2A, S2B |
| SWF147 | *flvEx76*[*ceh-36(prom3)*p::*GtACR2*::*T2A*::*gfp* + *myo-3*p::*mCherry*] | Gift from Steven Flavell | 2B |
| PY11012 | *oyEx658*[*odr-1*p::*GCaMP3* + *srsx-3*p::*mScarlet* + *unc-122*p::*dsRed*] | This paper | 2C, 2D, 4F, 4H, S2D, S2E, S4-2E |
| CX15257 | *kyEx5128*[*gcy-28d*p::*GCaMP5A* + *unc-122*p::*dsRed*] | (Larsch et al., 2015) | 3B, 3C, 4G, 4H, S3B, S3C, S4-2F |
| ZC2467 | *yxEx1364*[*ser-2(2)*p:: *FRT*::*STOP*::*FRT*::*GCaMP6s* + *odr-2b(3a)*p::*nFLP* + *unc-122*p::*dsRed*] | This paper | 3D, 3E, S3D |
| CX15261 | *kyIs617*[*gcy-28d*p::*HisCl1*::*SL2*::*gfp* + *myo-3*p::*mCherry*] | (Cho et al., 2016) | 3F |
| PY12206 | *oyEx659*[*ser-2(2)*p::*FRT*::*STOP*::*FRT*::*HisCl1*::*SL2*::*mCherry* + *odr-2b(3a)*p::*nFLP* + *unc-122*p::*gfp*] | This paper | 3G, S3F |
| CX17432 | *kyEx6105*[*ins-1(s)*p::*Chrimson*::*SL2*::*mCherry* + *elt-2*p::*mCherry*]; *kyEx5128*[*gcy-28d*p::*GCaMP5A* + *unc-122*p::*dsRed*] | (Lopez-Cruz et al., 2019) | 3H |
| CX17524 | *kySi77*[*FRT* before *eat-4* start codon] *kySi76*[*let-858UTR*::*FRT*::*mCherry* after *eat-4* stop codon] III; *kyEx6142*[*odr-1*p::*nFLP*] | (Lopez-Cruz et al., 2019) | 3I |
| PY12200 | *ins-1*(*oy158*)[*loxP*::*ins-1*::*loxP*] | This paper | 4A |
| PY12201 | *ins-1(oy158)*; *oyEx660*[*ins-1*p::*nCre*::*SL2*::*gfp* +*unc-122*p::*dsRed]* | Injected into PY12200 | 4A |
| PY12202 | *ins-1(oy158)*; *oyEx661*[*ifb-2*p::*nCre*::*SL2*::*gfp* +*unc-122*p::*dsRed*] | Injected into PY12200 | 4A |
| PY12203 | *ins-1(oy158); oyEx662*[*gcy-28d*p::*nCre*::*SL2*::*gfp* +*unc-122*p::*dsRed*] | Injected into PY12200 | 4A |
| PY12204 | *ins-1(oy158); oyEx663*[*odr-2b(3a)*p::*nCre*::*SL2*::*gfp* +*unc-122*p::*dsRed*] | Injected into PY12200 | 4A |
| PY11612 | *oyIs90*[*ins-1*p::*gfp*::*PEST* + *unc-122*p::*dsRed*] | This paper | 4B, S4-1C |
| CX7155 | *ins-1*(*nr2091*) | (Pierce et al., 2001) | 4D |
| DR1572 | *daf-2*(*e1368*) | CGC | 4D |
| DR26 | *daf-16*(*m26*) | CGC | 4D |
| RB712 | *daf-18*(*ok480*) | CGC | 4D |
| PY12207 | OH14125 [*ot853*[*daf-16*p::linker::*mNeonGreen*::*3xflag*::*AID*]]; *oyEx664*[*ceh-36prom2_del1ASE*::*TIR1*::*SL2*::*mTurquiose2 + unc-122p*::*dsRed + N2 gDNA*] | Injected into OH14125 | 4E |
| PY11614 | *ins-1*(*nr2091*); *oyEx658*[*odr-1*p::*GCaMP3* + *srsx-3*p::*mScarlet* + *unc-122*p::*dsRed*] | CX7155, PY11012 | 4F, 4H, S4-2E |
| PY11615 | *ins-1*(*nr2091*); *kyEx5128*[*gcy-28d*p::*GCaMP5A + unc-122*p::*dsRed*] | CX7155, CX15257 | 4G, 4H, S4-2F |
| PY12208 | *oyEx665*[*srg-47*p::*HisCl1*::*SL2*::*mCherry* + *unc-122*p::*gfp*] | This paper | S2C |
| CX13440 | *kyEx4018*[*inx-1*p::*GCaMP3* + *unc-122*p::*dsRed*] | (Gordus et al., 2015) | S3A |
| CX14599 | *kyEx4747*[*gcy-28d*p::*unc-103(gf)*::*SL2*::*mCherry* +*elt-2*p::*mCherry*] | (Cho et al., 2016) | S3E |
| PY12210 | GS8685 [*unc-119(ed3)*; *heIs105*[*rps-27*::*loxP*::*nls*::*mCherry*::*let-858UTR*::*loxP*::*nls*::*gfp*::*let-858UTR*]]; *oyEx674*[*gcy-28d*p::*nCre+gcy-28d*p::*cfp+unc-122*p::*dsRed*] Line 1 | (Ruijtenberg and van den Heuvel, 2015; Tenen and Greenwald, 2019); This paper | S4-1B |
| PY12211 | GS8685 [*unc-119(ed3); heIs105*[*rps-27*::*loxP*::*nls*::*mCherry*::*let-858UTR*::*loxP*::*nls*::*gfp*::*let-858UTR*]]; *oyEx674*[*gcy-28d*p::*nCre+gcy-28d*p::*cfp+unc-122*p::*dsRed*] Line 2 | (Ruijtenberg and van den Heuvel, 2015; Tenen and Greenwald, 2019); This paper | S4-1B |
| PY12212 | GS8685 [*unc-119(ed3); heIs105*[*rps-27*::*loxP*::*nls*::*mCherry*::*let-858UTR*::*loxP*::*nls*::*gfp*::*let-858UTR*]]; *oyEx675*[*odr-2b(3a)*p::*nCre+odr-2b(3a)*p::*cfp+unc-122*p::*dsRed*] Line 1 | (Ruijtenberg and van den Heuvel, 2015; Tenen and Greenwald, 2019); This paper | S4-1B |
| PY12213 | GS8685 [*unc-119(ed3); heIs105*[*rps-27*::*loxP*::*nls*::*mCherry*::*let-858UTR*::*loxP*::*nls*::*gfp*::*let-858UTR*]]; *oyEx675*[*odr-2b(3a)*p::*nCre+odr-2b(3a)*p::*cfp+unc-122*p::*dsRed*] Line 2 | (Ruijtenberg and van den Heuvel, 2015; Tenen and Greenwald, 2019); This paper | S4-1B |
| PY12214 | GS8685 [*unc-119(ed3); heIs105*[*rps-27*::*loxP*::*nls*::*mCherry*::*let-858UTR*::*loxP*::*nls*::*gfp*::*let-858UTR*]]; *oyEx673*[*ifb-2*p::*nCre + unc-122*p::*dsRed*] Line 1 | (Ruijtenberg and van den Heuvel, 2015; Tenen and Greenwald, 2019); This paper | S4-1B |
| PY12215 | GS8685 [*unc-119(ed3); heIs105*[*rps-27*::*loxP*::*nls*::*mCherry*::*let-858UTR*::*loxP*::*nls*::*gfp*::*let-858UTR*]]; *oyEx673*[*ifb-*2p::*nCre + unc-122*p::*dsRed*] Line 2 | (Ruijtenberg and van den Heuvel, 2015; Tenen and Greenwald, 2019); This paper | S4-1B |
| RB759 | *akt-1*(*ok525*) | CGC | S4-2A |
| TJ1053 | *age-1*(*hx546*) | CGC | S4-2A |
| JT9609 | *pdk-1*(*sa680*) | CGC | S4-2A |
| PY11616 | OH14125 [*ot853*[*daf-16*p::linker::*mNeonGreen*::*3xFlag*::*AID*]]; *oyEx666*[*srg-47*p::*TIR1*::*SL2*::*mTurquiose2 + unc-122*p::*dsRed + N2 gDNA*] Line 1 | Injected into OH14125 | S4-2C |
| PY11617 | OH14125 [*ot853*[*daf-16*p::linker::*mNeonGreen*::*3xFlag*::*AID*]]; *oyEx667*[*srg-47*p::*TIR1*::*SL2*::*mTurquiose2 + unc-122*p::*dsRed + N2 gDNA*] Line 2 | Injected into OH14125 | S4-2C |
| RB1341 | *nlp-1*(*ok1470*) | CGC | S4-2D |
| RB799 | *npr-11*(*ok594*) | CGC | S4-2D |
| MT15434 | *tph-1*(*mg280*) | CGC | S4-2D |
| FX02411 | *gcy-28*(*tm2411*) | NBRP | S4-2D |
| PY10808 | *ins-32*(*tm6109*) | NBRP | S4-2D |
| PY10712 | *ins-26*(*tm1983*); *ins-35*(*ok3297*) | PY10708, RB2412 | S4-2D |

S1-1: Figure 1 - figure supplement 1, S1-2: Figure 1 - figure supplement 2

S2: Figure 2 – figure supplement 1

S3: Figure 3 – figure supplement 1

S4-1: Figure 4 – figure supplement 1, S4-2: Figure 4 – figure supplement 2

**REFERENCES**

Cho, C.E., Brueggemann, C., L'Etoile, N.D., and Bargmann, C.I. (2016). Parallel encoding of sensory history and behavioral preference during *Caenorhabditis elegans* olfactory learning. eLife *5*, e14000.

Gordus, A., Pokala, N., Levy, S., Flavell, S.W., and Bargmann, C.I. (2015). Feedback from network states generates variability in a probabilistic olfactory circuit. Cell *161*, 215-227.

Hawk, J.D., Calvo, A.C., Liu, P., Almoril-Porras, A., Aljobeh, A., Torruella-Suarez, M.L., Ren, I., Cook, N., Greenwood, J., Luo, L.*, et al.* (2018). Integration of plasticity mechanisms within a single sensory neuron of *C. elegans* actuates a memory. Neuron *97*, 356-367

Larsch, J., Flavell, S.W., Liu, Q., Gordus, A., Albrecht, D.R., and Bargmann, C.I. (2015). A circuit for gradient climbing in *C. elegans* chemotaxis. Cell Rep *12*, 1748-1760.

Lopez-Cruz, A., Sordillo, A., Pokala, N., Liu, Q., McGrath, P.T., and Bargmann, C.I. (2019). Parallel multimodal circuits control an innate foraging behavior. Neuron *102*, 407-419

Pierce, S.B., Costa, M., Wisotzkey, R., Devadhar, S., Homburger, S.A., Buchman, A.R., Ferguson, K.C., Heller, J., Platt, D.M., Pasquinelli, A.A.*, et al.* (2001). Regulation of DAF-2 receptor signaling by human insulin and *ins-1*, a member of the unusually large and diverse *C. elegans* insulin gene family. Genes Dev. *15*, 672-686.

Ruijtenberg, S., and van den Heuvel, S. (2015). G1/S inhibitors and the SWI/SNF complex control cell-cycle exit during muscle differentiation. Cell *162*, 300-313.

Tenen, C.C., and Greenwald, I. (2019). Cell non-autonomous function of *daf-18*/PTEN in the somatic gonad coordinates somatic gonad and germline development in *C. elegans* dauer larvae. Curr Biol *29*, 1064-1072.
