# Supplementary material for: Feeding state functionally reconfigures a sensory circuit to drive thermosensory behavioral plasticity
Source: eLife. 2020 Oct 19;9:e61167. doi: 10.7554/eLife.61167 (PMC7644224; doi:10.7554/eLife.61167)
Supplement: Supplementary file 2. [file elife-61167-supp2.docx]

**Supplementary Table 2.** Plasmids used in this work.

| **Plasmid** | **Sequences** | **Promoter length** | **Source** |
| --- | --- | --- | --- |
| PSAB1201 | *odr-1*p::*GCaMP3* | 1.0 kb | This paper |
| PSAB1203 | *srsx-3*p::*mScarlet* | 1.3 kb | This paper |
| PSAB1204 | *odr-1*p::*HisCl1*::*SL2*::*mCherry* | 1.0 kb | This paper |
| PSAB1205 | *srg-47*p::*HisCl1*::*SL2*::*mCherry* | 650 bp | This paper |
| PSAB1206 | *ser-2(2)*p::*FRT*::*STOP*::*FRT*::*HisCl1*:: *SL2*::*mCherry* | 4.7 kb | This paper |
| pWY016 | *ser-2(2)*p::*FRT*::*STOP*::*FRT*  ::*GCaMP6s* | 4.7 kb | This paper |
| PSAB1207 | *ins-1*p::*nCre*::*SL2*::*gfp* | 4.2 kb | This paper |
| PSAB1208 | *gcy-28d*p::*nCre*::*SL2*::*gfp* | 2.8 kb | This paper |
| PSAB1209 | *odr-2b(3a)*p::*nCre*::*SL2*::*gfp* | 441 bp | This paper |
| PSAB1210 | *ifb-2*p::*nCre*::*SL2*::*gfp* | 3.0 kb | This paper |
| PSAB1211 | *ins-1*p::*gfp*::*PEST* | 4.2 kb | This paper |
| PSAB1212 | *srg-47*p::*TIR1*::*SL2*::*mTurquoise2* | 650 bp | This paper |
| PSAB1239 | *gcy-28d*p::*nCre* | 2.8 kb | This paper |
| PSAB1240 | *odr-2b(3a)*p::*nCre* | 441 bp | This paper |
| PSAB1241 | *ifb-2*p::*nCre* | 3.0 kb | This paper |
| PSAB1242 | *gcy-28d*p::*cfp* | 2.8 kb | This paper |
| PSAB1243 | *odr-2b(3a)*p::*cfp* | 441 bp | This paper |
| pWY046 | *odr-2b(3a)*p::*nFLP* | 441 bp | This paper |
| pUA118 | *ceh-36prom2_del1ASE*p::*TIR1*::  *SL2*::*mTurquoise2* | 1.6 kb | Gift from Oliver Hobert |
