# Supplementary material for: Feeding state functionally reconfigures a sensory circuit to drive thermosensory behavioral plasticity
Source: eLife. 2020 Oct 19;9:e61167. doi: 10.7554/eLife.61167 (PMC7644224; doi:10.7554/eLife.61167)
Supplement: Peer review history

# Decision letter

**DOI:** 10.7554/eLife.61167.sa1

**Affiliations:** Howard Hughes Medical Institute, Columbia UniversityUnited States; Yale University School of MedicineUnited States

In the interests of transparency, eLife publishes the most substantive revision requests and the accompanying author responses.

Thank you for submitting your article "Feeding state functionally reconfigures a sensory circuit to drive thermosensory behavioral plasticity" for consideration by *eLife*. Your article has been reviewed by three peer reviewers, one of whom is a member of our Board of Reviewing Editors, and the evaluation has been overseen by K VijayRaghavan as the Senior Editor. The following individual involved in review of your submission has agreed to reveal their identity: Daniel A Colón-Ramos (Reviewer #2).

The reviewers have discussed the reviews with one another and the Reviewing Editor has drafted this decision to help you prepare a revised submission.

As you will see below, all three reviewers agree on the importance and impact of this study and they agree that after a number of revisions this manuscript should become acceptable for publication. To summarize the reviewers comments and requested revisions:

Reviewer #1 asks for a simple control of the specificity of the Cre driver lines, which can be done with a simple cross.

Reviewer #3 has a number of editorial/data analysis suggestions that should be considered. This includes showing data preceding and following the temperature ramp (this data should be already available). Among the experimental suggestions, the key issue to address is to run some trials under isothermal conditions.

Reviewer #3 specifically requests better integration of previous literature.

Reviewer #1:

In this very nice paper the authors clarify and much extend previously documented effects of starvation in thermotaxis behavior. While previous work had established that starvation does affect thermotaxis, the precise cellular mechanisms had remained murky, at best. The authors use here a series of very rigorous and definitive experiments to show that intestinally produced insulin operates via an "accessory" sensory neuron, AWC, to modulate thermotaxis behavior. While described to some extent in other context, the extent and depth by which the author characterize this brain-gut signaling axis is unprecedented. Not only are the conclusions very interesting, the manuscript is very well written and clearly presented.

My only major quibble, which can be easily fixed, is that the elegant Cre/lox-mediated knockout of ins-1, very rigorous in principle, is not well enough described, particularly in regard to the negative controls. The authors state: "Knocking out ins-1 in all or a majority of ins-1-expressing cells, but not in AIA or AIZ/AIB, was sufficient to restore negative thermotaxis in starved animals (Figure 4A)." This sentence needs to be extended to state exactly what drivers were used to knock-out ins-1 where. The figure shows which cells were targeted, but does not show the driver. I would also like to see the specificity of these drivers (incl. the intestinal driver) being tested by an available "tester" strain in which a ubiquitous rfp transgene is excised, allowing expression of a gfp reporter (*heIs105* from Van der Heuvel, Cell, 2015 paper). Based on unpublished lore, Cre is extremely effective even at very low concentrations and spurious expression of Cre drivers can result in very misleading results.

Reviewer #2:

This is an elegant study by Takaishi et al. that investigates the effect of starvation on thermotaxis behaviors to address the broader conceptual question of how internal states alter behaviors. They use an array of cutting edge methods in imaging, genetics and behavioral analyses to identify a gut-brain signaling axis that modulates thermotaxis behavior based on starvation states. Their analyses achieve circuit dissection of an orthogonal circuit that modulates the core thermotaxis behavioral circuit. Their dissection achieved single-neuron, and sometimes, single neurotransmitter/neuromodulator resolution. The manuscript is significant in its findings, the experiments are well performed, and it is clearly written. It is an excellent piece of scholarship that will contribute significantly to the field.

Reviewer #3:

This study shows that effects of starvation on *C. elegans* thermotaxis behavior involve changes in the activity of sensory neurons and interneurons that are not components of the core thermotaxis circuit. The authors find that starvation increases the activity of AWC sensory neurons and decreases the activity of AIA and AIZ interneurons. At the core of the study is the observation that effects of starvation on thermotaxis can be bypassed with direct manipulations of AWC or AIA activity. These compelling observations are bundled with functional imaging data and a set of experiments suggesting that gut-derived insulin-like peptides trigger the observed changes in AWC and AIA activity. The study's strengths include the use of rigorous and quantitative behavioral and cell physiological methods, elegant genetic methods for conditional inactivation of genes, and very striking data showing that the activity states of AIA and AWC neurons determine thermotaxis behaviors. The study does, however, have some weaknesses. Throughout the manuscript, a statistic is used to describe neural activity that does not clearly capture all the relevant information about a neuron's activity. Also, the authors repeatedly describe neural activity as 'temperature responses' without data showing pre-stimulus activity or controls showing activity under isothermal conditions. These issues recur throughout the manuscript. The authors' interesting analysis of the effects of mutating the insulin signaling pathway on thermotaxis should be extended to include canonical effectors of the insulin receptor DAF-2. And last, the phenomenon under study deserves some discussion – the authors describe a remarkable effect of starvation on behavior but do not clearly explain what purpose it might serve.

• Earlier studies of AIY (Clark et al., 2006) indicate that calcium signals in these neurons faithfully follow those in AFD sensory neurons. It is not clear why in this study the authors see less faithful activation of AIYs (Figure 1F) by stimuli that consistently activate AFDs. The authors should explain this discrepancy.

• It is not clear that 'total response duration per neuron' is the best way to describe thermoresponses. This statistic does not capture the magnitude of a neuron's response, which probably matters as much as the duration of that response. The authors should analyze their data of neuronal responses in a way that also considers the magnitude of responses. This comment applies to multiple figure panels.

• The authors' claim that fasting increases the temperature responses of AWCs (subsection “The AWC olfactory neurons integrate feeding state information into the thermotaxis circuit”, last sentence) requires a control showing that under isothermal conditions there is no difference in the activity of AWCs in fed and starved animals. It remains possible that feeding-state-dependent changes in neural activity are independent of temperature.

• Along the same line, the authors' discussion of AIA responses describes them as temperature responses, but there are no data from before or after the temperature ramp shown, nor are there data showing neural activity under isothermal conditions. As shown, these data do not support the claim that these cells are responding to temperature stimuli. This comment applies also to imaging data from ins-1 mutants shown in Figure 4.

• More analysis of the insulin signaling pathway is warranted. The authors should test whether loss of genes encoding *daf-2*effectors, e.g. *age-1* and *pdk-1*, show the same behavioral phenotype as *daf-2* mutants.

• The authors' model predicts that fasting will not affect the activity of AIA interneurons in animals that either lack AWC neurons or whose AWC neurons are defective for insulin signaling. This should be tested.

• The authors' discussion of their study does not clearly put forth explanations of why thermotaxis and feeding state are coupled as they are. Is there some benefit to shutting down thermotaxis behavior when starved? Does thermotaxis occur at the expense of chemotaxis used to find food? This interesting phenomenon deserves some speculation.

# Reply: Author response

**DOI:** 10.7554/eLife.61167.sa2

Reviewer #1:

[…] My only major quibble, which can be easily fixed, is that the elegant Cre/lox-mediated knockout of ins-1, very rigorous in principle, is not well enough described, particularly in regard to the negative controls. The authors state: "Knocking out ins-1 in all or a majority of ins-1-expressing cells, but not in AIA or AIZ/AIB, was sufficient to restore negative thermotaxis in starved animals (Figure 4A)." This sentence needs to be extended to state exactly what drivers were used to knock-out ins-1 where. The figure shows which cells were targeted, but does not show the driver.

We have now indicated the exact drivers used for each cell and tissue type both in the main text of the Results, as well as in the Figure 4A legend.

I would also like to see the specificity of these drivers (incl. the intestinal driver) being tested by an available "tester" strain in which a ubiquitous rfp transgene is excised, allowing expression of a gfp reporter (heIs105 from Van der Heuvel, Cell, 2015 paper). Based on unpublished lore, Cre is extremely effective even at very low concentrations and spurious expression of Cre drivers can result in very misleading results.

This is an important control and we thank the reviewer for the suggestion. To address this issue, we expressed Cre under *ifb-2* (intestine), *gcy-28d* (AIA) and *odr-2b(3a)* (AIB and AIZ) promoters in the *heIs105* recombination reporter strain. To allow for the identification of Cre-expressing neurons, we also expressed CFP under the *gcy-28d* and *odr-2b(3a)* promoters. As shown in a new Figure 4—figure supplement 1B, we observe robust GFP expression in the gut but not in neurons in strains expressing *ifb-2*p*::Cre*. Conversely, we observe co-expression of CFP and GFP in AIA and AIB/AIZ interneurons in strains expressing *gcy-28d*p*::Cre* and *odr-2b(3a)*p*::Cre*, respectively. The positions of the soma and axonal trajectories of these interneurons are consistent with their cell identification.

Reviewer #3:

• Earlier studies of AIY (Clark et al., 2006) indicate that calcium signals in these neurons faithfully follow those in AFD sensory neurons. It is not clear why in this study the authors see less faithful activation of AIYs (Figure 1F) by stimuli that consistently activate AFDs. The authors should explain this discrepancy.

AIY responses are different depending on the type of thermal stimulus that is used for activation. Clark et al., 2006, as well as other papers including those from our lab (Biron et al., 2006, Matsuyama and Mori, 2020) use a rising oscillatory temperature stimulus. AIY responses are time-locked to these stimuli (and correlated with AFD responses at least in some temperature ranges – see Matsuyama and Mori, 2020). In contrast, AIY responses are more probabilistic and follow AFD responses less faithfully in response to a linear rising temperature stimulus, particularly in the *T>T_c_* or negative thermotaxis behavioral range (also see Hawk et al., 2018). The AIY calcium signals reported in this work in response to a linear rising temperature stimulus resemble those in the Hawk et al. paper (although we note that we use a shallower temperature ramp to more closely mimic the temperature changes experienced by animals navigating the thermal gradients in our behavioral assays).

• It is not clear that 'total response duration per neuron' is the best way to describe thermoresponses. This statistic does not capture the magnitude of a neuron's response, which probably matters as much as the duration of that response. The authors should analyze their data of neuronal responses in a way that also considers the magnitude of responses. This comment applies to multiple figure panels.

We describe three metrics for the calcium signals in each imaged neuron type: total response duration per neuron, average response duration per neuron, and response probability. Since with the exception of AFD calcium signals, temperature responses in all other examined neurons are stochastic, it is difficult to meaningfully quantify response amplitude. We also note that small differences in response amplitude can be artifactual, since response amplitude can be quite significantly affected by the expression level of the sensor.

• The authors' claim that fasting increases the temperature responses of AWCs (subsection “The AWC olfactory neurons integrate feeding state information into the thermotaxis circuit”, last sentence) requires a control showing that under isothermal conditions there is no difference in the activity of AWCs in fed and starved animals. It remains possible that feeding-state-dependent changes in neural activity are independent of temperature.

• Along the same line, the authors' discussion of AIA responses describes them as temperature responses, but there are no data from before or after the temperature ramp shown, nor are there data showing neural activity under isothermal conditions. As shown, these data do not support the claim that these cells are responding to temperature stimuli. This comment applies also to imaging data from ins-1 mutants shown in Figure 4.

In response to these very valid comments, we have now added a new Figure 2D and Figure 2—figure supplement 1D showing that AWC neuron responses are similar in fed and starved animals when held at a constant temperature of 20°C. We have also included a new Figure 3—figure supplement 1B and added new data to Figure 3—figure supplement 1C, showing that similar to the AWC neurons, AIA interneurons also do not exhibit differences in their calcium signals in fed and starved animals when held at a constant temperature of 20°C.These findings are consistent with our observation that the movement of fed and starved animals is largely indistinguishable under isothermal conditions, and is altered only on a thermal gradient (new Figure 1—figure supplement 1A).

• More analysis of the insulin signaling pathway is warranted. The authors should test whether loss of genes encoding daf-2 effectors, e.g. age-1 and pdk-1, show the same behavioral phenotype as daf-2 mutants.

Behavioral data from *akt-1, age-1* and *pdk-1* mutants are shown in Figure 4—figure supplement 2A.

• The authors' model predicts that fasting will not affect the activity of AIA interneurons in animals that either lack AWC neurons or whose AWC neurons are defective for insulin signaling. This should be tested.

While these are excellent suggestions, these are complex experiments to perform technically, and are challenging to complete under the current restrictions. We hope that the behavioral experiments demonstrating the role of glutamatergic signaling from AWC (Figure 3I) together with additional behavioral and imaging experiments described here support our model sufficiently.

• The authors' discussion of their study does not clearly put forth explanations of why thermotaxis and feeding state are coupled as they are. Is there some benefit to shutting down thermotaxis behavior when starved? Does thermotaxis occur at the expense of chemotaxis used to find food? This interesting phenomenon deserves some speculation.

We speculate on this briefly at the end of the first paragraph in the Discussion. In short, based on our previous observations that prolonged starvation increases olfactory responses in AWC (Neal et al., 2015), we propose that the altered sensory responses in AWC allow animals to prioritize food-finding over optimal thermoregulation.
